# “Time to Change”: To What Extent Could Non-Sputum Sampling Accelerate the Fight Against Tuberculosis—A Qualitative Study Among End-Users

**DOI:** 10.3390/tropicalmed10020044

**Published:** 2025-02-05

**Authors:** Vanessa Fargnoli, Hoang Thi My Hanh, Ly Na Hoang, Ananya Mahesh, Nasiphi Mqedlana-Ntombela, Jovitta Xavier, Mikashmi Kohli, Kavindhran Velen, Sonjelle Shilton

**Affiliations:** 1 FIND, 1218 Geneva, Switzerland; mikashmi.kohli@finddx.org (M.K.); kavindhran.velen@finddx.org (K.V.); sonjelle.shilton@finddx.org (S.S.); 2 Health Strategy and Policy Institute (HSPI), Hanoi 100000, Vietnam; hoangmyhanh@hspi.org.vn; 3 FIND, Hanoi 100000, Vietnam; lyna.hoang@finddx.org; 4 Resource group for Education and Advocacy for Community Health (REACH), Chennai 600014, India; ananya@reachindia.org.in (A.M.); xmjovi@yahoo.co.in (J.X.); 5 The Aurum Institute, Johannesburg 2194, South Africa; nntombela@auruminstitute.org

**Keywords:** tuberculosis, diagnosis, non-sputum sampling, urine, tongue swabs, end-users, qualitative study

## Abstract

Inadequate access to timely diagnosis and linkage to treatment are major barriers to tuberculosis (TB) care. New point-of-care diagnostics that do not rely solely on sputum samples are needed to make up for lost time, bringing TB testing closer to service recipients and addressing current sputum sampling limitations. Urine-based TB lipoarabinomannan tests and tongue dorsum swabs have demonstrated potential as alternatives to sputum-based molecular testing. We conducted a study to ascertain the perceived value of these non-sputum-based TB tests among stakeholders from the TB community, including TB service recipients and healthcare providers, in India, South Africa, and Viet Nam. Our results showed that there was a high degree of enthusiasm among various end-users for both novel sample types. It is important to generate both qualitative and quantitative evidence to support optimal uptake and implementation of these potential new sample types for TB testing.

## 1. Introduction

Tuberculosis (TB) remains a major global health threat, the “greatest infectious killer” [1]. According to the World Health Organization (WHO), TB is one of the top-ten causes of death and the leading cause of single infection [2]. Each year, TB afflicts approximately 10 million people and causes 1.5 million deaths, despite the existence of effective treatment [2] that means it is a curable disease. Inadequate access to timely diagnosis and linkage to treatment represent considerable barriers to TB care; this situation was exacerbated by the COVID-19 pandemic [3].

Sputum and bronchial alveolar lavage samples are currently the primary WHO-approved sample types for TB diagnosis and the main sample types used for diagnosis [4,5]. However, reliance on sputum-based testing is a problem because it hampers case-detection efforts, particularly among individuals with paucibacillary TB (e.g., people living with HIV, children) and those investigated earlier in their TB disease progression, for whom sputum production is inherently difficult [6,7]. These issues mean that sputum-based diagnostics can often leave TB service recipients without a confirmed diagnosis. Furthermore, it can take many days to weeks before an individual learns about their TB test result. There can often be at least four touchpoints, including sample collection at a decentralized facility, sample transport to a centralized laboratory, returning the results to the decentralized facility, and contacting individuals to give them their results. This prolonged time from testing to diagnosis increases the risk of individuals being lost to follow-up and is a major contributor to poor TB outcomes [8]. These limitations have prompted endeavors to find alternative sample types that are simpler, less intrusive, and safer to collect [3,6].

New point-of-care (POC) diagnostics that do not rely solely on sputum samples are needed to make up for lost time, bring testing closer to service recipients, and address current sputum sampling limitations. Urine-based TB lipoarabinomannan (LAM) tests can be completed at the POC in just 25 min and have been shown to decrease the time to a confirmed diagnosis and treatment initiation, resulting in reduced mortality among service recipients with concurrent TB and HIV co-infections [9]. However, the diagnostic tool has relatively poor sensitivity and is only recommended for people living with HIV with severe TB symptoms and/or advanced HIV. Budget limitations and a lack of local operational research studies to inform implementation have also been reported [10]. The Fujifilm SILVAMP TB-LAM test (FujiLAM, Fujifilm, Tokyo, Japan) is part of the next generation of urine-based TB diagnostics. It takes 50 min to complete, has increased sensitivity compared with other tests, and shows promise for HIV-neutral populations, although it is not currently available on the market. Tongue dorsum swab testing has demonstrated potential as an alternative to sputum-based molecular testing in terms of the speed, simplicity, and safety [6,11]. Moreover, the ease of collecting tongue dorsum swabs from individuals in diverse settings makes it particularly useful for contact tracing and community-based screening purposes [6], as summarized in Table 1

Each of these novel diagnostics has primarily been evaluated in eastern and southern Africa, where the HIV prevalence is among the highest in the world. However, there are limited data available on preferences in relation to novel sample types for TB diagnostics in low- and middle-income countries (LMICs) [7]. Two studies, one conducted in Malawi and Zambia, and the other in Kenya, Mozambique, and South Africa, assessed the urine-based Fujifilm SILVAMP TB-LAM test [7,10]. These studies found that the urine-based diagnostic was acceptable to both service recipients and healthcare workers, as it increased the ease and convenience of TB testing by removing the need for sputum collection. The rapid results (which could be delivered to clients in 50 min) were believed to increase access to TB diagnostics and reduce the loss to follow-up by removing the need for service recipients to return to a clinic for their results. Healthcare workers considered the test was easy to use and required minimal training; however, they were concerned that the urine-based diagnostic could negatively affect the clinical workflow [7]. While these findings increase our understanding of end-user perceptions of urine-based TB diagnostics, the findings may not apply to other new sample types, such as tongue swabs. Codsi et al. (2022) assessed facilitators and barriers in relation to the use of experimental tongue-swabbing methods (11), as reported by healthcare workers in South Africa. The main facilitators reported were an aversion to sputum samples, the perceived safety of the method, and the availability of educational resources to train patients, while the main barriers reported were cultural stigma, personal security, control of the work environment, and the risk of COVID-19 [11].

There has been little qualitative research into TB diagnostics [12]. However, it has been reported that decisions about TB care are often made without considering end-user needs [3], especially TB service recipients and healthcare providers. Empirical evidence about patients’ needs and pathways to diagnosis [12] is essential to inform diagnostics manufacturers and decision- and policy-makers. Therefore, to strengthen the evidence base regarding the preferences for non-sputum sample types among intended end-users, we conducted a multi-country study in India, South Africa, and Viet Nam, which rank among the top-30 countries with the highest burden of TB in the world.

In 2020, India had a TB incidence of 188 per 100,000 population and a mortality rate of 37 per 100,000 population. Although the country has been actively involved in TB control activities for more than 50 years, TB continues to be India’s most severe health problem [13]. India now has a target to eliminate TB by 2025; thus, there is a need for increased diagnosis and treatment of TB, along with improved intersectoral coordination. Currently, TB diagnosis relies heavily on sputum examination for pulmonary TB detection.

South Africa has the eighth highest TB incidence rate in the world, with 554 people living with TB per 100,000 population in 2020. In 2019, 58,000 South Africans succumbed to TB [2]. Challenges with sputum collection and the time taken for clients to receive confirmed TB results have undermined progress. The country would benefit from alternative sampling and testing strategies that would improve the TB screening process and is actively seeking innovative diagnostic strategies to reduce the burden on TB services and poor TB outcomes.

Viet Nam ranks 10th out of 30 countries with the highest TB burden and is placed 11th out of 30 countries with the greatest burden of multiple drug resistance (MDR). Half of all TB cases in Viet Nam are not identified by general symptom-based screening and are identified by chest X-ray [14]. A reliance on sputum-based testing has hampered case-detection efforts [15], particularly among individuals with paucibacillary TB infection and those investigated earlier in their TB disease progression, for whom sputum production is inherently difficult. New, fit-for-purpose people-centric tests are thus urgently needed to enable wider and more equitable access to TB testing.

The aim of the present study was to ascertain from end-users their perceptions regarding the non-sputum sample types and to understand the potential barriers and facilitators when integrating these new sampling methods into existing TB care pathways. In the three countries where we conducted the study, we targeted four end-user groups: (1) TB service recipients, (2) civil society (CS) stakeholders, (3) healthcare workers (HCWs), and (4) public health (PH) experts. We sought to identify the arguments for and against the potential implementation of urine and tongue swab samples and pave the way for a policy-based discussion. The data were collected at a time when neither of these sample types were validated or recommended for TB testing globally or in-country.

## 2. Materials and Methods

The results reported here are qualitative findings based on open-ended survey questions and semi-structured interviews that formed part of a larger mixed-methods study (the results of the mixed-methods study are reported in a separate manuscript). Prior to data collection, the interview guides were reviewed and piloted by the three study teams to ensure the clarity of the questions and their cultural appropriateness.

Participants were identified using purposive and opportunistic sampling strategies. The inclusion criteria for all the groups included in the study were age (adults aged 18 years or more) and the ability to speak at least one local language. An additional inclusion criterion for the TB service recipients was that they either needed to be evaluated for TB or were currently living with TB. For the CS, HCW, and PH groups, an additional inclusion criterion was that they needed to be in a position where they engaged with members of the community and with TB testing.

The HCWs included certified personnel, lay cadres, and community volunteers who regularly interacted with TB service recipients, providing direct care, counseling, advocacy, or other forms of support, or were health facility-based laboratory technicians. The CS stakeholders were individuals who played a key leadership or coordination role in their community and/or TB advocacy/representation. The PH experts were individuals or members of organizations who had valuable insights or inputs regarding how TB diagnostics function and local experiences with TB diagnostics.

The TB service recipients received compensation to complete the survey. This incentive was ZAR 300 (approximately USD 16.12) in South Africa, INR 1000 (approximately USD 12.82) in India, and VND 100,000 (approximately USD 4.08) in Viet Nam. Additionally, in Viet Nam, the semi-structured interview participants were compensated with VND 200,000 (approximately USD 8.16). All the surveys and semi-structured interviews were conducted in relevant local languages (Vietnamese, Hindi, IsiZulu, Setswana, Sesotho, or English), either face-to-face or remotely (via Teams, Zoom, or phone). With participants’ permission, all the interviews and open-ended questions were audio-recorded.

**Figure A1:**
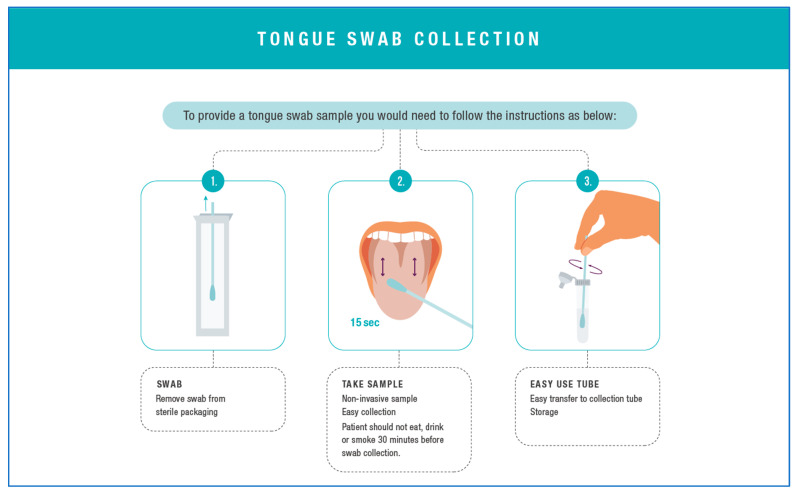
Tongue swab for recipients of care.

**Figure A2:**
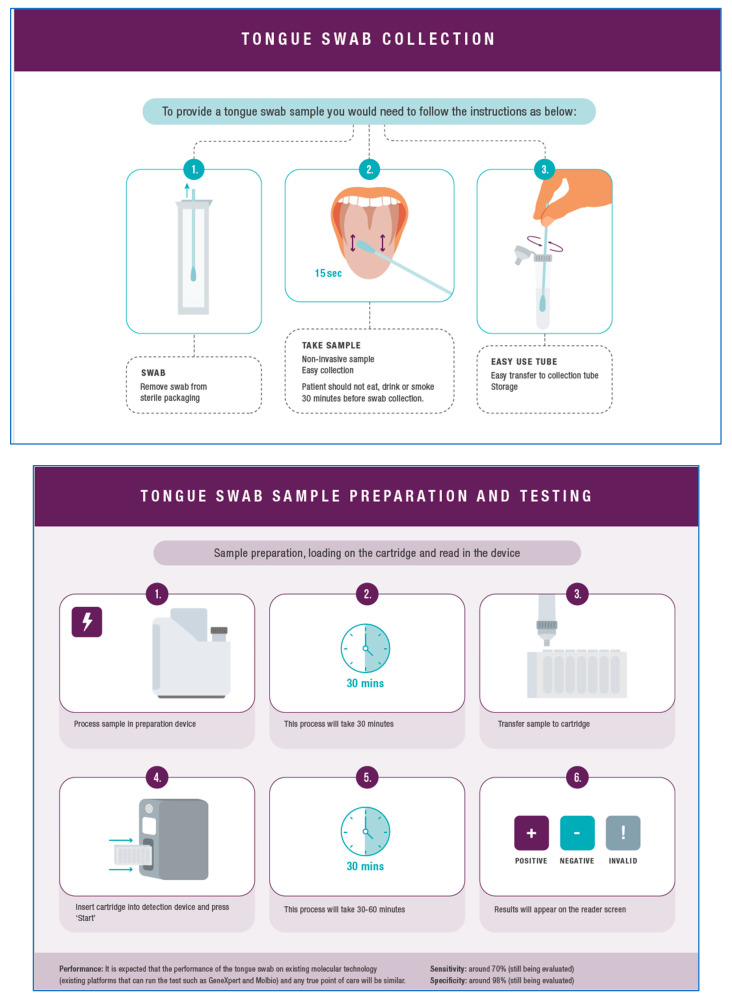
Tongue swab for health care providers.

**Figure A3:**
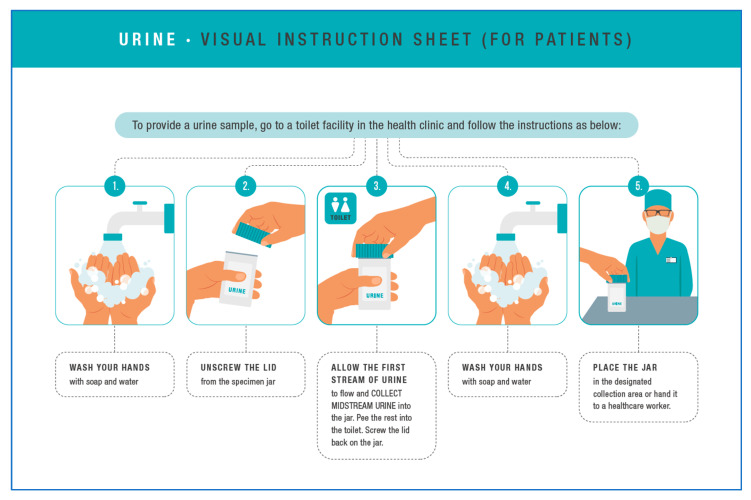
Urine sample for recipients of care.

**Figure A4:**
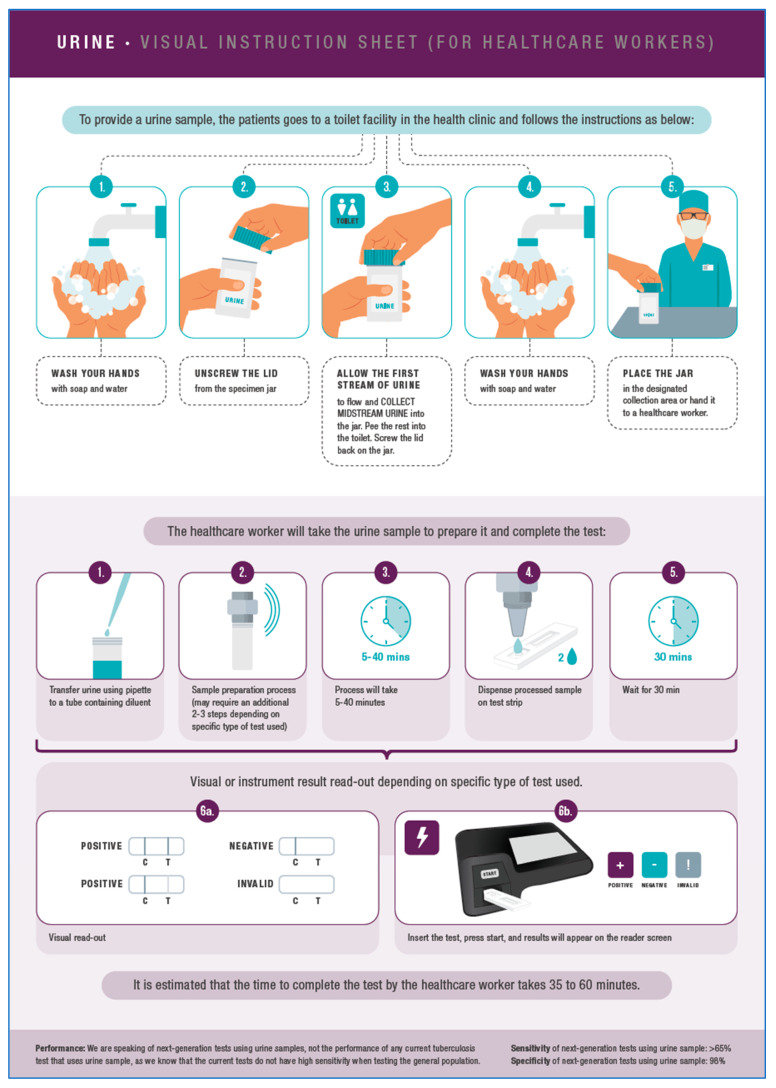
Urine sample for health care providers.

To assist participants in envisioning the use of these novel sample types within their own real-life settings, we created visual information sheets. One set of explanatory materials was aimed at TB service recipients and provided a plain-language overview of the novel sample types, while a second set of explanatory materials, for the CS, HCW, and PH stakeholders, included more technical details (visual information sheets, Appendix A, Figure A1, Figure A2, Figure A3 and Figure A4).

This study and the related study documents were approved by the Research Ethics Review Committee of the WHO (ERC.0003871) and by national ethics review committees in Chennai, India (Resource group for Education and Advocacy for Community Health, independent ethics committee), Johannesburg, South Africa (University of the Witwatersrand), and Hanoi, Viet Nam (Hanoi University of Public Health, institutional ethical review board, 96/2023/YTCC-HD3). All the participants provided written informed consent to participate in the study, and all the data were anonymized prior to analysis.

The data were analyzed using thematic content analysis [16,17]. For the open-ended answers (OEAs), we used consensual qualitative research-modified (CQR-M) analysis [18]. The interviews and open-ended answers provided redundant opinions. We therefore considered having reached data saturation and completed the fieldwork in June 2023.

The interviews and open-ended answers were fully transcribed and, for those conducted in local languages, translated into English. The interview transcripts were then systematically coded using Quirkos CAQDAS v2.5.3 software (list of codes, Appendix B, Table A1). The coding process for the interview transcripts was conducted in two steps: (1) the interview guide topics provided the initial codes, and (2) some inductive codes were created based on the collected data. We held regular meetings with the three study teams, during which we identified common themes by country and by question.

## 3. Results

Between March and June 2023, we conducted 62 semi-structured interviews (32 interviews with HCWs, 15 with CS stakeholders, and 15 with PH experts). For the OEAs based on the completeness of answers, 4407 answers were analyzed out of 5560 transcripts from a total of 898 surveys (470 surveys with TB service recipients, 307 with HCWs, 60 with CS stakeholders, and 61 with PH experts). There were almost twice as many transcripts from Viet Nam (n = 1752) and India (n = 1695) as from South Africa (n = 960).

In total, 960 participants were enrolled in this study: 505 women and 455 men, in 54 sites: 14 in India (4 in Muzaffarpur, 2 in Vaishali, Bihar state; 4 in Champwat, 4 in Haridwar, Uttarakhand state), 16 in South Africa (8 in Ekurhuleni district, Gauteng province and 8 in Bojanala district, North West province), and 24 in Viet Nam (7 in Hanoi, 6 in Da Nang, 5 in An Giang province, and 6 in Ho Chi Minh).

More precisely, 470 TB service recipients participated in this study, with median ages of 32, 37, and 46 years in India, South Africa, and Viet Nam, respectively. In South Africa, the majority of respondents were women (n = 86), while in Viet Nam, the majority were men (n = 110). In India, there was gender parity. Respondents lived in urban (n = 226), peri-urban (n = 71) and rural (n = 173) areas. In India and Viet Nam, more than half of the TB service recipients received no or a primary education. In South Africa, the majority had received a secondary education (n = 120). Less than half of the TB service recipients were unemployed, especially in India and South Africa, while few were employed or on a daily wage, particularly in Viet Nam and India (Table 2).

The semi-structured interview respondents held various professional positions and had experience at different facility levels (Table 3).

Their age ranged from 27 to 70 years, and half of them were women. The majority worked in an urban setting, and their experience of working in TB services ranged from 1 to 38 years. The interviews lasted, on average, 86 min in Viet Nam and 40 min in South Africa and India.

The participants’ experiences with sputum samples are briefly outlined below, followed by their opinions of the new sample types and the implications of their impact and implementation for TB care systems. We present the results of the data for the three countries and for the groups jointly, except when there is a specificity or a disparity between countries and groups.

To preserve the participants’ anonymity, we only specify the following information after a quote: the country (India, SA for South Africa, and VN for Viet Nam), the participant group (TB rec for TB service recipients, HCW, CS, or PH), gender (W for woman and M for man) (all participants identified themselves as either women or men), and the age rank (Y for “young”, i.e., 18–30 years; M for “middle-aged”, i.e., 31–60 years; and S for “senior”, i.e., 61 years or more).

### 3.1. Experiences with Sputum Samples

When asked about their previous experiences with sputum sampling, the TB service recipients expressed mixed feelings. For example, respondents expressed varied opinions regarding the comfort and ease of sputum sample collection. In Viet Nam and South Africa, the majority reported no difficulties, while in India, most of the TB service recipients stated they had experienced a lot of difficulties (n = 109). Globally, more women than men expressed that they had experienced difficulties in producing a sputum sample. In South Africa and India, the fear of receiving a positive result and the negative consequences of having a TB-positive status were often highlighted.

Every time a person comes to test, they have the shock! They get anxious. How will I feel if I have this type of disease? Do you understand? It’s not easy. (SA, TB rec, W, Y)

For most HCWs and TB experts, the lack of early identification of TB and subsequent delayed treatment initiation due to the prolonged times taken to process samples and return results remained key challenges associated with sputum samples:

I want improvement in TB services. It should consume less time for patients. Get tested quickly and treatment can start immediately to reduce infection spread. (India, HCW, M, M)

At the systemic level, the importance of “patient counseling”, including support and comprehensive TB information provided at the individual and community level, was mentioned by all the stakeholders. This means that the relationship between healthcare providers and service recipients was considered important to strengthen:

Your day at work may be a lifetime experience for your patients. As a healthcare provider, it might be just one of those days at work, but for your patient, it is one day that sort of meant everything in their life. It’s important to care about the experience of our patients. (SA, CS, M, M)

The risk and fear of TB infection were also discussed. To prevent and reduce this risk, all the stakeholders emphasized the need for counseling and training of various individuals, including health professionals and cleaning staff, as well as ensuring there is a sufficient stock of protective equipment, such as masks, gloves, and disinfectants. Many respondents reported recurrent stock-outs, leading them to adopt alternative strategies:

Gloves are not available. I use masks and sanitize my hands. Then, I use the plastic cover that we usually receive with the container and wrap it again with another plastic cover. Then, I take the sample and give it to the center. (India, CS, W, Y)

Most respondents called for “new” TB tests. This leads us on to our inquiries regarding the participants’ perspectives on the proposed new sample types.

### 3.2. Opinions on Urine Samples and Tongue Swabs

The perceived benefits and challenges regarding the two proposed sample types compared with sputum samples, as well as their perceived infection risk and their acceptability, are discussed below.

#### 3.2.1. Perceived Benefits

When we asked the participants for their opinions regarding tongue swabs and the urine LAM test, the responses were very similar for both sample types. The three main advantages suggested were “easy and simple procedures”, “convenient and comfortable for patients” (we use the term patients when it refers to the opinions of the participants), and “time saving” for HCWs and TB service recipients (with HCWs’ workload reduced and linkage to care increased):

Easy to understand the diagnosis and [people] can be put on treatment easily. This can be done faster. (India, HCW, W, M)

Some considered tongue sampling to be more hygienic and cleaner compared with urine or sputum sample collection:

It [tongue swab] is clean. You don’t have to look at the sputum or urine or fecal matter. Relative to all the other tests, the tongue swab stands out as one easy to do. (SA, PH, M, M)

South African stakeholders found that the ease of tongue swab collection could be an advantage as other individuals, such as community health workers (community health workers are lay healthcare workers employed to carry out TB contact tracing within communities in South Africa), could use test kits during their field visits:

What I really like, it [tongue swab] would be an easier test for a community health worker to perform. (SA, CS, M, M)

In India, both sample types were seen as an advantage for people living in remote areas, as they could be diagnosed and treated on the same day.

When patients come from mountainous areas, they spend a lot of time traveling, they would like all the tests related to diagnosis to be performed in a day. (India, HCW, M, M)

For the urine sample type, self-sampling at home and the potential for self-testing were considered to be an important advantage for TB service recipients, given the lack of hygienic toilets in some healthcare centers and mass-screening camps, particularly in Viet Nam and India.

The collection within the facility might pose a lot of challenges, providing the water or hand sanitizer. There is no gender specific in most of the OPSs [outpatient services] unless it is clinic or hospital. They may not be able to facilitate the process of urine collection. By providing an option for home-based collection that would be an important thing. (India, CS, M, M)

The reduced risk of infection that HCWs would face was perceived as a benefit for tongue self-sampling, followed by providing people with empowerment, freedom, and choice.

It [self-sampling] will reduce the risk of spreading significantly. Self-sampling is so simple, it doesn’t have to be a lot of spit. If I can use this self-sampling test kit, the risk of infection will be reduced. (VN, CS, W, M)

If this tongue swab does not require too much technical, it’s not too difficult. It should be left to the patients to take. (VN, HCW, W, M)

The familiarity with urine and tongue swab samples was seen as a benefit by most HCWs:

We do it on a daily basis for a lot of other diagnoses, even the urine LAM, the one that we used to test TB, we also embark on that! (SA, HCW, W, Y)

Some CS and PH respondents considered the tongue swab sample type to be particularly suitable for community screening:

The advantage is that the sampling time is fast and suitable for all subjects, from the elderly people to young children, people with sputum or not. (VN, CS, M, M)

For both sample types, respondents acknowledged that they were easier and more convenient for groups in which sputum samples are difficult to collect, such as children, pregnant women and the elderly, who are also perceived to be priorities in the fight against TB:

The challenge, especially with pregnant women, is that they want to produce the sputum but they don’t have it! If there was a way we could find TB without having to produce sputum that is actually sensitive, that will be great! (SA, HCW, W, Y)

[Detecting] Pediatric TB is one of the biggest weaknesses of the national TB program, and this happens not only in Viet Nam but also to the national TB program of other countries. (VN, PH, W, M)

For some Vietnamese and Indian HCWs, urine sampling was assessed as “only good” for these groups and people living with HIV and should not be generalized to the entire population.

Some concerns about stigmatization and discrimination were raised in all three countries, mainly among participants engaged in community-based activities. Both sample types were described as good for reducing stigma as they are not associated with TB alone. Tongue swabs were regarded as the best option to avoid discrimination, as individuals would not need to leave the consulting room or visit the restroom; they could remain within the privacy of the consultation area, away from the gaze of others.

This one [tongue] is better because it doesn’t discriminate anyone and then there is no need for the patient to go outside. Once I am assessing this woman, it’s just to get the consent from her and then I do the swab and then it is done, rather than for the patient to go to the toilet and collect the sputum and come back. (SA, HCW, W, M)

The ability to perform the tests as part of general and routine practice was also perceived to be beneficial in avoiding stigma:

If you take a sputum sample, people will identify it as tuberculosis. But the urine is a routine test, diabetic tests are also done through urine sample, so people will not mind giving the sample. (India, CS, W, Y)

#### 3.2.2. Perceived Challenges

Conversely, the “low accuracy of the results” and “doubts about detecting TB in urine or tongue”, both of which could lead to missed diagnosis or unnecessary treatment, were cited as the two main disadvantages of these sample types.

It should not give false-positive samples, then it would create problems and affect patients. If a person does not have TB but this test declared him/her positive, treatment will begin and this would affect the health. (India, HCW, M, M)

TB is a lung disease. The origin of TB is the lungs. We are going to have missed diagnoses if we rely on it [new sample] to diagnose TB. (SA, HCW, M, M)

Urine cannot contain TB bacteria. (VN, TB rec., M, M)

The lack of availability of sanitary facilities, especially for women, was perceived as a major gender-based barrier for urine samples:

The biggest problem is the toilet. The toilets of the health facilities are very dirty. There is no place to get them. I just had the check-up last weekend. The men’s restroom is open, the women’s restroom is locked, the female patients can’t get urine! (VN, CS, W, M)

There are no bathrooms available, and if it is available, the person may or may not let the person use it. (India, PH, M, M)

Based on the TB service recipients’ perceptions, tongue swab samples were considered more suitable for everyone compared with the suitability of urine samples, which were considered to be a more “complicated and less hygienic” procedure, especially in India and Viet Nam.

I find it [urine sample] harder to get! It’s inconvenient to have to go to the toilet. It is difficult to get it into the jar. (VN, TB rec, M, M)

The midstream urine requirement was considered to be an issue for pregnant women and the elderly because of the difficulty they might have in controlling the flow of urine:

You urinate and then catch up the second urine. It’s so difficult for pregnant women because once they want to pee, they want to pee everything. This one [midstream urine] is very challenging. (SA, HCW, W, M)

Midstream [urine] is the biggest problem. (VN, CS, W, M)

#### 3.2.3. Risk of Infection

Tongue swabs were perceived to be more risky than urine in terms of contamination but less risky than sputum:

Sputum is worse because a person must cough! There must be that force pressure. So, it’s easy for droplets to go out and have a contact with whoever, another patient. (SA, CS, W, M)

Sputum will spread to others; however, the tongue swab poses less chances of spread. (India, CS, W, M)

Sneezing and coughing were frequently highlighted as potential reactions to tongue swab sampling, putting HCWs at risk of exposure to TB:

If I take this sample directly and they have a reaction, they sneeze, and I will have a higher risk of infection. (VN, HCW, W, Y)

The risk of infection associated with urine sampling was considered low, or even non-existent, compared with sputum, especially in settings with limited aerosol containment.

The risk of infection is almost zero. Technology detects DNA or structural changes in cells in urine. The presence of live TB bacteria is the one to be at risk of infection. (VN, PH, W, M)

Only in South Africa was the processing of urine samples considered to be risky. However, a specific risk associated with urine samples was noted by various stakeholders and was related to toilets, a shared closed space where TB service recipients could infect others by coughing when collecting their urine specimen:

Urine itself is not contagious, but here you are concerned that in a closed environment, people might cough or sneeze. (VN, HCW, W, Y)

#### 3.2.4. Acceptability

The acceptability of the new sample types was evaluated based on respondents’ feelings of (dis)comfort and (dis)trust, and on their reported experiences with sputum samples, categorized as either painful or not painful, as well as if they did not receive a result back at any stage in the sputum testing process.

##### Comfort

Overall, all the participants assessed the new sample types as being comfortable. To improve TB service recipients’ comfort, health professionals and experts recommended enhanced patient counseling and HCW training and the provision of clean, hygienic, private, and separate rooms in all health facilities. These factors were considered essential for both sample types:

Counseling is key because people are very aware about this kind of test [tongue], this is not new! Counseling is very important. (India, PH, M, M)

There is a need to organize a training course for healthcare workers, especially sample collectors, on how to counsel patients so that we have knowledge and skills to advise patients. (VN, HCW, M, Y)

To ensure urine sampling could be comfortably conducted, separate toilets for men and women were deemed an essential gender consideration in India and Viet Nam. This was emphasized by both women and men respondents:

In every health center, separate toilets should be available for female patients to collect the sample easily. (India, HCW, W, M)

Gender- and user-friendly urine containers, adapted to each morphology for women’s and men’s bodies, were recommended by healthcare workers. Furthermore, these containers should be of high quality and made of opaque material, so as not to see the urine:

The sampling tubes should be designed differently for different genders, which will bring comfort for patients. (VN, HCW, M, M)

The interviewees were also concerned about TB service recipients’ ability to collect midstream urine and understand the instruction to do so, considering this to be almost as difficult and inconvenient as collecting sputum:

I’m skeptical with the instruction. It nearly looks like a sputum. It is not easy to collect sputum! So, this is nearly like the issue of sputum, you know the midstream one! (SA, CS, W, M)

[If] you have to collect urine midstream and the patient has already urinated. Not everyone who sits for a while can urinate! (VN, PH, M, M)

Here, 20 mL of urine required, but some just peed 5 mL. Males have it easier than females in collecting urine. Running urine is the biggest issue. (VN, CS, M, Y)

Some stakeholders added that women would feel less comfortable than men collecting a urine sample in public toilets due to the lack of hygiene and maintenance and also related to the urine sample container:

Females may be uncomfortable with public toilets. Some females opt not to go to a public facility bathroom for hygienic purposes. They may generally be more negatively impacted than males. (SA, PH, M, M)

It’s far from easy for women to collect running urine in this bottle. (VN, CS, M, Y)

At the time of this study, South Africa was experiencing load and water shedding. This means that during these periods, no water and/or electricity were available, creating an additional inconvenience for urine sample collection:

Sometimes, we don’t have water or there is a load shedding, and then you ask somebody to go the bathroom! That woman needs to wash her hands. I think the infection part of it will be compromised. When we have load shedding, it won’t work for us. (SA, HCW, W, M)

In India and South Africa, some HCWs found that the 30 min delay required to receive the results of a urine sample test was too long for TB service recipients:

It’s a longer period of time to wait. If it was like the HIV test, which takes 15 [minutes], it would be ideal if the time for urine test reading could be shortened. (SA, CS, M, M)

In India and South Africa, some HCWs believed that collecting urine samples would consume too much of their time, with a risk of increasing their workload.

Due to the risk of infection with tongue swab sampling, open, well-ventilated, and air-conditioned rooms were seen as essential for the safety and comfort of TB service recipients and HCWs. It was also important to consider the tongue swab design, making them soft and high quality, to improve their comfort.

All the stakeholders unanimously stressed the importance of providing specific instructions on whether TB service recipients should avoid eating and drinking before tongue sampling, and on the time they should wait after eating/drinking/smoking to take the sample, to ensure samples are collected properly. This was referring to the risk of having “contaminated” samples that could interfere with the results:

The mouth is forever contaminated! People eat this and that, so depending on the time when the swab is collected and the procedure prior to collecting the swab, I foresee contaminated test results or contaminated samples. (SA, HCW, M, M)

For urine samples, the risk of having a “contaminated” sample due to chemicals or medications was only raised in Viet Nam and for the elderly:

Elderly people aged from 60 and over use about 11 drugs on a daily basis. From vitamins to bone and liver tonics. Because this is a color indicator, it will interfere with the results. (VN, PH, M, S)

As mentioned earlier, the ability to self-test with a tongue swab and/or urine sample was perceived to be a potential benefit and a way to improve TB service recipients’ comfort.

If the test is performed by a rapid kit as used in case of pregnancy and malaria tests, the patient has to put two drops and the test will be conducted automatically, that will be easy! (India, HCW, M, M)

The CS and PH respondents believed that HCWs would be comfortable with both sample types because of the ease of the procedures and the time saved:

When the patients come to get medicines, the urine test can be administered to them on the same day, and the report can be given immediately too. It is easy for both of them [HCW and patients]. (India, CS, M, Y)

X-ray is easier, so sputum in hospitals, it’s not performed compared to X-ray. You check the advantages of the [tongue swab] compared to the one we are using. You’ll see. They [nurses] will take the easiest one. It can even replace [other tests]. (SA, CS, W, M)

According to the Indian CS and PH respondents, most laboratories are well equipped to handle urine samples, which could make HCWs more comfortable with and accepting of using these samples:

Someone is going to process the urine sample in an automated apparatus. You just load it in the cartridge or extraction device. It’s going to be a simple procedure for everybody, which can even be kept at district microscopic centers. (India, PH, W, M)

However, some stakeholders also reported that HCWs dislike working with stool or urine samples and therefore might be reluctant to adopt this new sample type for TB diagnosis. To improve HCWs’ comfort with urine samples, the majority of experts stressed the need to increase the accuracy of results and simplify the procedures, i.e., to reduce the number of steps involved in urine testing, in particular the concentration steps, and reduce the amount of consumables used.

It counts several steps! Six steps for the results. The pipette test involves a pipette tube, but the tube is a problem, not to mention two or three further steps required for the preparation of specimen. Too many steps discourage our medical workers, especially at the district level! [And] fewer accessories or consumables. For a test that requires 20 accessories versus one with only 3, you will prefer the one with 3! (VN, PH, W, M)

In Viet Nam, some respondents recommended that HCWs’ working conditions be improved, notably through financial compensation, while in South Africa, they recommended increasing human resources and training.

##### Trust

The reasons mentioned by the TB service recipients for trusting the results of urine and/or tongue samples were correlated with the ease of the procedure, including privacy, the opportunity for self-sampling, and the time saved, i.e., receiving the results the same day:

This test is performed directly in the mouth. It is easier. Wake up and get tested! As simple as that! (India, TB rec, M, M)

The tongue swab, I like it because it brings privacy. It takes out the results fast. It’s easy to use it! (SA, TB rec, W, M)

They believed that the quality of the samples and the accuracy of the results were better than with sputum samples. This also relates to the fact that by “seeing” the processing, individuals can be confident that any results belong to them:

They [urine tests] have been performed in front of you. The other ones [sputum] go to the lab, they go for days. What if they exchange or make mistakes there? It is better! It’s performed in front of you, there is no mistake, no exchange so you trust them because you saw when they do their things in front of you. (SA, TB rec, W, Y)

Tongue swabs were also seen as being more advanced and therefore “more trustable” compared with sputum, which was considered old.

In Viet Nam, some TB service recipients mentioned that they trusted HCWs’ advice and recommendations from the Ministry of Health. They would therefore follow this advice regardless of the sample type; this was also highlighted by the Vietnamese CS and PH respondents (a manuscript addressing the role of HCWs in the Vietnamese context is currently in preparation).

I believe in the results if it is tested, verified, and recommended by healthcare workers. (VN, TB rec, M, M)

They will do whatever the doctor says, so the most important thing is good advice from the medical staff. (VN, CS, W, Y)

Familiarity with urine sample and tongue swab testing for other diseases was also a key reason for trusting these new sample types.

Conversely, were the several reasons given by the TB service recipients who stated they had less trust in the results obtained from urine and/or tongue swab samples compared with the results from sputum samples. These were the low accuracy of the results (sensitivity too low), doubts about the ability to detect TB in urine or tongue swab samples (among young and senior participants), the lack of trust in a test that has never been used before, and their familiarity with sputum sampling:

This is new so I don’t know much. The sputum sampling is still a traditional method, so I trust it more. (VN, TB rec, M, M)

I can’t trust the results. Cough comes from lungs. My problems are in my chest. Since cough comes from my chest, I will prefer sputum sample. (India, TB rec, W, Y)

According to the CS and PH respondents, trust could be improved through various means. These include patient counseling, providing scientific evidence, the use of gender- and user-friendly sampling devices and tubes, simplification of the procedure, integration of these new tests into the routine testing procedure, and via community awareness campaigns or “propaganda”:

Only propaganda can do it! There must be propaganda to explain the effectiveness of the test so that medical staff can believe and propagate. (VN, CS, M, M)

Sometimes, our issues are failing because we are not taking correct information to communities. Communities need to be brought on board. They should know what is happening. If they know what is happening, it will be easy. (SA, CS, W, M)

From the perspective of the CS and PH respondents, HCWs’ trust in the new sample types is linked to the accuracy of the test results, the ability to immediately receive the results, and the reduced risk of infection. The advantages of saving time, including the ability to detect diseases other than TB, and the familiarity with tongue swab and urine tests (e.g., for COVID-19, diabetes, and pregnancy), were often quoted as factors influencing trust, particularly in India and South Africa:

They have already performed this routine during the COVID-19 period. Therefore, this will be like a general practice for them. (India, CS, M, M)

Some perceived that if tests based on tongue swab or urine samples had a low sensitivity, they could be acceptable for community screening but not at the clinic level:

The sensitivity is relatively low, 70%, that could be sort of acceptable if it’s at a community level, but at a clinic level we will be missing a lot of TB! It might be something that could be used, let’s say, for people who are really unable to produce sputum in a health facility and then also at the household level. (SA, CS, M, M)

Finally, some stakeholders suspected that the results from tongue swab samples would not be trusted, as saliva is not considered to be a “good sample”:

We do not consider “spit” as a good sample. (India, CS, W, Y)

### 3.3. Impact on and Implementation Within Existing TB Care Systems

During the interviews, we also assessed the potential for the use of urine or tongue swab sampling to resolve the challenges associated with sputum sampling. Delays in receiving results, repeated sampling due to invalid sputum samples leading to frequent returns to clinics, and individuals facing difficulties or pain when trying to produce a sputum sample were common reasons quoted for the loss-to-follow-ups highlighted by both experienced and less-experienced respondents. They perceived that urine and tongue swab sampling could lead to increased adherence to screening and treatment, thus reducing the spread of infection within communities.

Most of the dropout cases are because of difficulties in giving sputum as a sample, but with this test, they can give the sample without any hesitation and treatment can be started, thus it will decrease the dropout rate. (India, HCW, M, M)

What we want is to ensure that more people are tested for TB so that we can diagnose them and get them on treatment. Or, if they don’t have TB and they’re high risk, offer them TB preventive therapy. It would be a big advantage to increase testing in the country to have a point-of-care diagnostic to scale up TB diagnosis and get people on treatment if they need it and then also to scale up TB prevent therapy for those who would be eligible. (SA, CS, M, M)

Testing numbers would increase significantly with these tests. If testing increased, more patients suffering from TB would be diagnosed. (India, HCW, W, Y)

During the interviews, the participants shared their opinions on how the urine and tongue swab samples could be implemented in their country. In India, the majority felt that the tongue swab and urine samples could be “easily” processed at primary healthcare centers (the findings of the study conducted in India will be reported in a separate manuscript that is currently in preparation). Some recommended they be introduced at health and wellness centers or at district TB centers (alluding to the fact that clean toilets are available at these locations), where the population load is high, to increase the coverage. Village health nurses and some experts from international health organizations felt that tongue swab collection could be performed at the village level by Accredited Social Health Activists (ASHAs) (ASHAs have a wide reach in the community, especially in rural areas):

If ASHA workers are trained, it would be very good since they go to rural areas where TB patients are diagnosed more in comparison to cities. (India, CS, W, Y)

In South Africa (the findings of the study conducted in South Africa are reported in a separate manuscript that is currently under review at BMC Health Services Research), sufficient equipment and human resources, stock availability, including tests kits, specimen bottles for urine samples, and protective equipment, were considered crucial to ensure successful implementation. Concerns were raised that tongue swab samples could produce inconclusive results, similar to COVID-19 tests, and that there could be a shortage of test kits:

From my previous experience from COVID-19, most of the results were inconclusive. As far as swabs go, I do not think they have accurate results. (SA, PH, M, M)

With the COVID-19 test, there would be times where simple things like the syringes for the vaccination were out of stock. How do you expect us to work without the resources? My concern is that there must be a regular flow of resources and protective gear. In that way, the service won’t be interrupted! (SA, HCW, W, M)

In Viet Nam, the experts agreed that the novel samples could be implemented at district health centers (for tongue swabs) and communal health stations (for urine samples), providing there was sufficient infrastructure and equipment and that staff were well trained.

If it is using the Xpert Gen System of that program, it is a daily operation” (VN, PH, W, M).

It’s equipment, machinery, and facilities! (VN, HCW, W, M)

In all the countries, the majority of HCWs believed that these sample types could easily be integrated into their routine testing practice:

It’s quicker! It will not affect us negatively. I work with [pregnant] women. When we collect blood, we can just include the one for swabbing and it will be quick. (SA, HCW, W, M)

Stakeholders from the three countries believed that using tongue swabs could help to further decentralize TB testing. Some even saw it as a “game changer” in TB testing:

What are they [manufacturers] waiting for when implementing this? Because these are some of the interventions, I mean innovations we want! We want innovation that would enable us to initiate patient early. (SA, PH, M, M)

When we asked the health professionals and experts what their recommendations would be to change and/or improve the proposed new sample types, they reiterated the need to improve their performance in terms of the results, which should be at least similar to sputum to be acceptable, especially among laboratory technicians:

The sensitivity of the test should be at least 90%, because the sensitivity of popular screening and diagnostic tests available on the market today are more than 90%. (VN, HCW, W, M)

Some respondents questioned, when using these sample types, their ability to detect or rule out individuals with TB who had only been showing symptoms for a few days:

We need to be able to pick it up soon, not when a person has already had symptoms for 1 or 2 months. We need to be able to suspect TB in someone who has had two days of cough and has risk factors for TB. We need to be able to rule it out. So I’m not sure that tongue swab will be able to pick up organisms at that stage compared to a sputum sample. (India, PH, W, M)

Tongue swabs were considered inappropriate for active case finding among the general community, as individuals who had experienced symptoms for a long period of time would usually be unwell and thus present in a hospital:

This [tongue test] would probably need someone with a longer duration of symptoms in order for the test to pick it up. (India, PH, W, M)

Concerns regarding the potential increase in HCWs’ workload were also raised, as a confirmatory test would still be needed to detect drug resistance. This was considered to be critical information necessary to initiate an appropriate treatment regimen and an essential aspect of the TB care cascade in all the countries concerned:

You still have to determine whether there is drug resistance or not before starting treatment so they still have to send them [patients] anyway. (VN, HCW, W, M)

I am not sure if it [tongue] could give us resistance and GeneXpert can, so GeneXpert is still indispensable in this situation. (SA, HCW, M, Y)

## 4. Discussion

In this study, we explored new approaches to non-sputum sampling when collecting samples for TB testing in India, South Africa, and Viet Nam. This study is strengthened by the inclusion of heterogeneous population groups, such as TB service recipients’ opinions, in various settings, including urban, peri-urban, and rural areas. Our findings thus provide new perspectives in the field of TB diagnosis.

The challenges related to sputum collection have been widely discussed in the literature [6,19,20]. Consistent with the challenges previously identified, participants in all three countries reported a lack of access to healthcare facilities suitably equipped for sputum sampling, especially in rural areas; difficulty in providing sputum samples, especially for children, the elderly, and pregnant women; a lack of timely diagnosis and linkage to treatment; the high rates of individuals being lost to follow-up; the risk of infection; and the stigma associated with TB. Most participants welcomed the two non-sputum sampling options and supported their implementation.

Similar to the findings from other studies [6,7,9,10,11,21,22], the advantages reported in favor of the new sample types were mainly related to the simplicity and ease of use of the sampling procedures in comparison with sputum sampling. These included the ease of sample collection; accessible, rapid results; convenience for individuals being tested; and the familiarity with tongue swab and urine tests, which were perceived as being easy to integrate into HCWs’ routine activities and contributing to enhanced patient outcomes. Non-sputum samples were considered suitable for key groups such as children, pregnant women, the elderly, and people living in isolated areas. The recurrent emphasis on these advantages implies that participants consider them to be key features, potentially influencing their adoption. Similar to Chenai Mathabire Rücker et al. (2022), the respondents in our study often noted the need for appropriate training and sufficient human and material resources. Having on-the-spot diagnostics that can provide same-day results is important if diagnostics are to be used to inform the management of TB [7] and, more broadly, other diseases [23]. Dissociating the sample type from the disease was considered by both the TB service recipients and the HCWs to be a major asset in terms of avoiding stigmatization. True POC testing, self-sampling, and self-testing were also perceived to be key advantages in tackling stigmatization and avoiding exposure to discriminatory behavior by HCWs.

Conversely, arguments made against the novel sample types stressed their low accuracy and inability to detect drug resistance. These were major concerns in the three countries where we conducted this study and have also been reported elsewhere [1,7]. The majority of respondents stipulated that the new tests should yield accuracy levels equivalent to existing diagnostic tests to be accepted and implemented. In line with Herrmann et al. (2022), the inability of children to urinate on demand was reported (7), with urine samples considered unsuitable for children by respondents in South Africa and Viet Nam but not in India.

Similar to the findings from other studies, the multiple steps required for the urine LAM test were seen as a challenge and a burden that added to HCWs’ workload [7,10]. The eligibility criteria for which individuals should be tested were not discussed, and it was assumed that all individuals presenting with symptoms would be tested based on the new samples, again leading to increased work for HCWs. Sufficient and proficient staffing both at healthcare facilities and in the field emerged as pivotal factors associated with the success of implementation. Furthermore, HCWs raised concerns regarding the hygiene of oral samples, in terms of the samples being “contaminated” with food or drugs.

The level of infrastructure and human resources could pose a major challenge to introducing the new sample types in India, South Africa, and Viet Nam. These countries have settings where resources are limited, including the limited availability of sanitary facilities, a critical issue for women specifically. Therefore, the urine LAM test could represent an additional gender-related barrier to women accessing diagnostics [24]. This is a contextual determinant that is important to address in LMICs and is often reported in India. As highlighted by Engel (2020), “technologies should be attuned to particular contexts of use” (12). This emphasizes the importance of promoting healthcare systems that are more responsive to gender-specific needs.

Irrespective of the sample type, comfort and trust were (apart from the performance of the testing tools) a question related to patient counseling. Respondents stated that it was essential to provide clear, detailed instructions for users and “good care”, which included providing a welcoming, safe, and clean environment within testing facilities. These factors were also seen as key facilitators of implementation. A study addressing what are community members’ beliefs and expectations regarding having TB is yet to be undertaken, as both the lay and experienced individuals’ concerns regarding the new sample types were based on the opinion that the detection of TB is only possible if the sample is obtained from the lungs. This has an impact on the trust in samples collected from sites other than the lungs.

Respondents considered it was important to address the risk of infection, regardless of the sample type, to protect HCWs and the population in general. The majority of respondents considered sputum collection to be the sampling method that posed the greatest risk of infection. It was also noted that the act of collecting a tongue swab sample may cause a patient to cough and thus present a risk of infection for healthcare staff. No risk perception relating to SARS-CoV-2 virus was suggested [11].

Self-sampling using tongue swabs emerged during discussions as being a safer practice for HCWs compared with sputum sampling. A preference for self-sampling to be conducted at a facility instead of at home was also reported by Codsi et al. (2022) (11). The location where urine samples are collected was perceived to represent a risk for individuals, as toilets are shared spaces where people with TB could infect others if they were coughing when collecting their urine specimens. To address the risk of infection, respondents in each of the three countries emphasized the need to train individuals attending healthcare settings, healthcare professionals themselves, and other staff working in healthcare settings, such as cleaners.

If the novel sample types could be integrated into existing TB testing services in the three countries, the use case for this integration would greatly depend on the tests’ performance, the level of infrastructure, human resources, and increased awareness among communities. Although our findings support further exploration of the use of tongue swabs and urine LAM tests, especially for groups who cannot produce sputum, there remains a need to further optimize both the technology (increase test sensitivity) and the system (increased awareness and agency, as well as gender and key group specificity and representation). Despite the high degree of enthusiasm for each of the novel sample types, the participants considered that they did not currently represent a suitable alternative with which to replace sputum sampling. Instead, they felt the new sample types could represent complementary tests in the TB testing pathway that would require further tests to be conducted, therefore increasing the complexity of the existing TB algorithm and testing routine.

There were some limitations to our study. Studies involving theoretical scenarios have limitations due to their hypothetical nature, as the applicability, performance and generalizability of theoretical tools may not always align with real-life contexts. Individuals may express preferences regarding the new sample types, but this does not guarantee that these individuals will actively use or be willing to accept the use of these new samples. Further studies are needed to understand the willingness to use new, alternative sample collection methods in real-world settings, and any potential consequences, to assess “user-technology interactions” [25].

There may have been some desirability bias in our study, as the surveys of TB service recipients took place in healthcare facilities. This may have impacted some of their responses regarding their experiences of the TB services. Some respondents may have chosen to give “safe” answers due to the theoretical nature of this study. Furthermore, some healthcare workers may have felt that they would have been affected professionally if they expressed certain concerns.

The preferences for tongue swab samples may have been influenced by the COVID-19 pandemic, the consequences of which were still present when this study was conducted and which was often spontaneously mentioned in the discussions and the examples provided. Finally, most of the participants were from the public sector; therefore, our findings might not reflect the reality in the private sector.

Despite these limitations, this study did have considerable strengths. Firstly, this study was conducted in multiple countries with a significant sample size; this allowed the findings to account for different health systems and testing approaches (and healthcare-seeking behavior), which may ultimately influence the adoption of new sample types and tools. Secondly, to our knowledge, this is among the first studies to consider earlier views on these new sample types to inform current development and future adoption of TB diagnostic tools. Lastly, the robust study design ensured that the data were broadly generalizable within the regions where this study was conducted.

## 5. Conclusions

This study provides a snapshot of opinions in relation to non-sputum sample types before their implementation in three countries with a high burden of TB. Despite being a theoretical study involving participants who had no experience of using the novel sample types for TB testing, our qualitative results offer a global understanding that enables us to foresee potential challenges and make recommends for decision-making for a subsequent phase of practical implementation. This will aim to ensure the adaptability of the new tools for diverse contexts and needs. Our findings improve our understanding not only of the technologies but also of the systemic issues and the way they are interrelated with diagnostic processes. Technologies may fail to achieve their goals if they do not integrate the social dynamics of health systems that are driven by individuals’ needs and experiences. The interviews show that the community and end-users are willing to adopt these platforms and sample types once we have more concrete and robust evidence. These findings could help advocate for an easier and more rapid uptake of such tests and sample types, as it was a systematic effort to assess peoples’ needs for people-centric decentralized tests.

Despite cultural, socio-economic, and contextual differences across the countries involved in our study, the end-users expressed common preferences and challenges. A major strength of this study was the deliberately chosen diversity, which enabled an in-depth exploration of the perceptions within populations characterized by their variable access to resources and led to preliminary results concerning how to develop fit-for-purpose samples with better people-centered diagnostics and care. Evidence on the use of the new sample types in context is crucial, and this study shows the importance of involving and engaging with various communities and actors to better understand the complexity of TB diagnosis processes.

## Figures and Tables

**Table 1:** Sample collection methods.

Test	Sample Type	Sample Collection	Point-of-Care Placement	Time to Result	Limit of Detection
Nucleic Acid Amplification Test (NAAT) ^§^	Sputum	Requires well-ventilated space for collection	No	±90 min	16 colony forming units [cfu]/mL
Urine LAM Test *	Urine	Requires designated ablution facilities	Yes	<30 min	500 pg/ml

^§^ Based on the GeneXpert MTB Ultra. * Based on the commercially available Determine TB LAM Ag.

**Table 2:** Sociodemographic characteristics of TB service recipients by country.

TB Service Recipients (n = 470)	India (n = 161)	South Africa (n = 154)	Viet Nam (n = 155)
Gender Men Women	80 81	68 86	110 45
Age (median) 18–25 26–45 46–60 >60	32 (24–51) 48 65 20 28	37 (32–46) 15 96 36 7	46 (32–57.5) 16 60 45 34
Education University Secondary Primary College None/no response	20 44 46 0 51	15 120 18 0 1	24 36 71 11 13
Employment Employed Self-employed Unemployed Daily wage labor Retired Student	14 2 82 50 1 12	52 10 81 1 7 3	40 32 33 28 13 9
Area of residence Urban Rural Peri-urban	82 72 7	33 58 63	112 43 0

**Table 3:** Summary of semi-structured interview participants’ characteristics by country.

Country	Stakeholder Group	Specific Role	Number of Participants Interviewed
India			
	HCW	Medical officer	2
		Senior treatment sup.	2
		Laboratory technician	2
		Village health nurse	2
		TB health visitor	2
	CS	State TB coordinator	1
		TB champion	4
	PH	Member of a national research organization	1
		Member of an international health organization	1
		State TB officer	1
		Private practitioner	1
		Member of a non-governmental organization	1
South Africa			
	HCW	Professional nurse	9
		Clinical doctor	2
	CS	TB advocacy group member	4
		TB champion	1
	PH	HIV/AIDS/STI/TB director	2
		Key official	2
		District/provincial TB director	1
Viet Nam			
	HCW	Testing department staff member	1
		Leader of a TB hospital	1
		Head/deputy head of a laboratory	3
		Head of a clinic	2
		Nurse	3
		Deputy head of a commune health station	1
	CS	Expert member of TB program	1
		Senior technical officer of an international non-governmental organization	1
		Public health association representative	1
		Researcher	1
		Technical expert at an international non-governmental organization	1
	PH	Representative of a public health institution	1
		Representative of an organization involved in guiding international funding in Viet Nam	1
		Representative of a tertiary hospital involved in TB care	1
		Representative of an INGO involved in TB research	1
		Representative of a TB program	1

## Data Availability

FIND does not share qualitative data due to the methodological challenges associated with secondary data analysis conducted by researchers not involved in the data collection and thus not able to conduct secondary data analysis while embedded in the context of the research.

## Appendix B. List of Codes

**Table A1:** Deductive codes for the three countries involved in this study.

Themes	Codes
TB testing (actual)	
Experience	TB testing experience
Risk of infection	TB sample risk of infection
HCW–patient relationships	
TB patient consideration	TB patient consideration
Tongue swab collection	
Acceptability of tongue swabs	Acceptability of using a tongue swab (personal)
	Acceptability of using a tongue swab (others)
Risk/safety collection tongue	Risk of infection tongue swab
Self-sampling	Client tongue swab self-sampling at a facility
	Client tongue self-sampling at home
Tongue swab integration	
Tongue swab integration and performance	Tongue sample integration in daily activities
	Tongue sample integration in TB testing
Tongue sampling at primary healthcare (PHC) facility	Tongue sampling at PHC feasibility
	Tongue testing at PHC feasibility all staff
Tongue swab finance	
Tongue swab cost coverage	Tongue swab cost coverage
Tongue swab acceptable price	Tongue swab acceptable price
Tongue swab implementation	
Tongue swab implementation in existing platforms key points or in a true POC platform key points	Tongue swab implementation
Impact on treatment	Treatment (tongue swab sample)
Impact on dropout rate	Loss to follow up rate (tongue swab sample)
Tongue swab support implementation	Support tongue swab implementation
Urine sample collection	
Urine sample collection location	Urine sample collection location
Acceptability of urine samples	Acceptability of using urine samples (personal)
	Acceptability of using urine samples (others)
Risk/safety collection	Risk of infection with urine samples
Urine sample integration	
Urine sample integration and performance	Urine sample integration in daily activities
	Urine sample integration in TB testing
Urine testing at a PHC facility	Urine testing at PHC feasibility
	Urine testing at PHC feasibility all staff
Urine sample finance	
Urine sample cost coverage	Urine sample cost coverage
Urine sample acceptable price	Urine sample acceptable price
Urine sample implementation	
Urine sample implementation	Urine sample implementation
Urine sample impact on treatment	Treatment (urine sample)
Impact on dropout rate	Loss to follow up rate (urine sample)
Urine sample support implementation	Support for urine sample implementation
Inductive codes by country	
India	
	Current challenges in TB testing
	Challenges in self-sampling
	Saving time (tongue swab)
	Saving time (urine sample)
	Self-testing (urine sample)
	Stigma
South Africa	
	Advantage of urine/tongue swab samples compared with sputum
	Experiences/lessons learned from the COVID-19 pandemic
	Main characteristics needed for tongue swab samples
	Main characteristics needed for urine samples
	Staff attitude
	Participant questions/recommendations
Viet Nam	
	Unique belief and trust of Vietnamese patients
	COVID-19-relatedness
	Reject urine sample
	Reject tongue swab sample
	TB end
	Mass screening
	Advantages of tongue swab samples compared with sputum
	Advantages of urine samples compared with sputum
	Tongue swab sample sensitivity expectation
	Urine sample sensitivity expectation
	Other benefits of tongue swab samples
